# Complete genome sequence of *Leeuwenhoekiella parthenopeia* H156, a potential carbohydrate polymer degrader isolated from tidal flat sediment in Zhejiang, China

**DOI:** 10.1128/mra.00934-25

**Published:** 2025-11-05

**Authors:** Wen-wu Zhang, Bo-xu Chen, Yue Su, Cong Sun

**Affiliations:** 1 College of Life Sciences and Medicine, Zhejiang Sci-Tech University 12646, Hangzhou, China; 2 Trend Biotech Co., Ltd, Hangzhou, Zhejiang, China; California State University San Marcos, San Marcos, California, USA

**Keywords:** *Leeuwenhoekiella*, tidal flat, polysaccharide degradation

## Abstract

We report the complete genome sequence of *Leeuwenhoekiella parthenopeia* H156 isolated from tidal flat sediment in Zhejiang, China. The genome consists of a 4.43 Mbp circular chromosome with annotated 331 carbohydrate-active enzymes. It contains 32.5 glycoside hydrolases/Mb, higher than other *Leeuwenhoekiella* genomes, indicating its potential in carbohydrate polymer degraders.

## ANNOUNCEMENT

Genus *Leeuwenhoekiella*, belonging to the family *Flavobacteriaceae*, has versatile metabolic capabilities for carbohydrate polymers (1), typically having a larger genome size (>4 Mbp) and diverse carbohydrate-active enzymes (CAZymes) (2). The genomic analysis of *Leeuwenhoekiella* strains can reveal the ecological roles and adaptation mechanisms of carbohydrate polymer degraders in tidal flats.

The sediment sample of the tidal flat was collected with a mud sampler from Taizhou, Zhejiang Province, People’s Republic of China (28°33′ N, 121°21′ E) in March 2023 and divided into 50 mL sterile sampling tubes after sampling. Twenty grams of these sediments was suspended in 50 mL sterile 2% NaCl solution (wt/vol) then diluted with sterile 2% NaCl solution (wt/vol) to a concentration of 10^−4^ and spread on 1/10 strength marine agar (BD). After 4 days at 30°C, one colony was picked and named strain H156. The genomic DNA was extracted using SDS extraction method as described (3).

After quality assessment, DNA samples were sent to Guangdong Magigene Biotechnology Co. Ltd. (China) for library construction using the ALFA-SEQ DNA Library Prep Kit (Findrop, China) and SQK-LSK109 kit (Oxford Nanopore). Resulting libraries were sequenced on the Illumina NovaSeq 6000 and Oxford Nanopore PromethION platforms (4), and the quality was controlled by Sickle v.1.33 (https://github.com/najoshi/sickle) and Mecat2 v.2.0 (https://doi.org/10.1038/nmeth.4432), respectively. The ONT sequencing data were basecalled by Dorado v.7.1.4 (https://github.com/nanoporetech/dorado).

The Illumina NovaSeq 6000 platform generated 6,345,042 paired-end reads with a read length of 150 bp, and the Oxford Nanopore PromethION platform generated 755,034 reads with an N50 value of 6,182 bp. After assembling with Unicycler v.0.4.8 (5), the genome quality was estimated by CheckM v.1.0.7 (6). The 16S rRNA gene of strain H156 was extracted with RNAmmer v.1.2 (7) and compared to other *Leeuwenhoekiella* members in the National Center for Biotechnology Information (NCBI) 16S Ribosomal RNA (Bacteria and Archaea Type Strains) database (updated data: 9 October 2025) using NCBI blastn v.2.14.1 (https://blast.ncbi.nlm.nih.gov/Blast.cgi). According to the minimum standard proposed by Riesco and Trujillo (8), the average nucleotide identity (ANI) was calculated by the ANI Calculator v.0.90 (https://www.ezbiocloud.net/tools/ani), and digital DNA-DNA hybridization (dDDH) values were estimated using the GGDC calculator v.3.0 (https://ggdc.dsmz.de/ggdc.php#) with BLAST+ and default parameters. The genomic annotation was conducted with RAST server v.1.3.0 (https://rast.nmpdr.org/) and dbCAN3 v.13 (https://bcb.unl.edu/dbCAN2/blast.php) and classified by the CAZy database v.3.4.7 (https://www.cazy.org/) (9). The prediction of polysaccharide utilization loci (PULs) was performed as described (10).

According to the assembly results of Unicycler, the genome of the H156 strain was a single circular genome in 4.43 Mbp with 42.1% G + C content. The genome contained three ribosomal RNA operons, 40 tRNA genes, and 3,782 coding DNA sequences. Analysis of the 16S rRNA gene sequence based on the EzBiocloud indicated strain H156 was most closely related to *Leeuwenhoekiella parthenopeia* Mr9^T^ with 99.9% similarity, and the dDDH and ANI values between them were 73.2% and 97.4%, respectively, indicating strain H156 should be assigned to *L. parthenopeia*.

Strain H156 encoded 331 CAZyme genes, its glycoside hydrolase (GH) density (32.5/Mb) exceeds other reported *Leeuwenhoekiella* genomes (20–25/Mb) (11). In addition, 13 PULs for degrading xylan, mannan, pectin, and fucoidan were predicted. Among them, xylan- and pectin-related CAZymes, such as GH10, GH28, GH39, GH43, GH67, CE1, CE8, CE12, PL1, PL9, and CBM9 were annotated (12, 13).

## Data Availability

The genome sequence of strain H156 has been deposited in the National Center for Biotechnology Information (NCBI) under accession number CP197499 with BioProject and BioSample accession numbers PRJNA1292446 and SAMN50000908, respectively. The raw data for the genome sequence of strain H156 have been deposited in the NCBI Sequence Read Archive database under accession numbers SRR34674729 and SRR34674730. The RAST and dbCAN3 annotation results and the PULs prediction results for strain H156 are publicly available in Zenodo.
